# Supplementary material for: ATP activation of peritubular cells drives testicular sperm transport
Source: eLife. 2021 Jan 27;10:e62885. doi: 10.7554/eLife.62885 (PMC7840184; doi:10.7554/eLife.62885)
Supplement: Transparent reporting form [file elife-62885-transrepform.pdf]

1st Floor
24 Hills Road
Cambridge CB2 1JP, UK

P 01223 855340
W elifesciences.org
T @elife

eLife's transparent reporting form

We encourage authors to provide detailed information *within their submission* to facilitate the interpretation and replication of experiments. Authors can upload supporting documentation to indicate the use of appropriate reporting guidelines for health-related research (see EQUATOR Network), life science research (see the BioSharing Information Resource), or the ARRIVE guidelines for reporting work involving animal research. Where applicable, authors should refer to any relevant reporting standards documents in this form.

If you have any questions, please consult our Journal Policies and/or contact us: editorial@elifesciences.org.

Sample-size estimation

- You should state whether an appropriate sample size was computed when the study was being designed
- You should state the statistical method of sample size computation and any required assumptions
- If no explicit power analysis was used, you should describe how you decided what sample (replicate) size (number) to use

Please outline where this information can be found within the submission (e.g., sections or figure legends), or explain why this information doesn't apply to your submission:

No statistical power analysis methods were used to predetermine sample size. Sample size was determined based on our previous experience and according to accepted practice in the field. All sample size information (cells / tubules / experiments (n)) are denoted in the respective figures and / or legends. General information is additionally found in the materials and methods section (subsection data analysis).

Replicates

- You should report how often each experiment was performed
- You should include a definition of biological versus technical replication
- The data obtained should be provided and sufficient information should be provided to indicate the number of independent biological and/or technical replicates
- If you encountered any outliers, you should describe how these were handled
- Criteria for exclusion/inclusion of data should be clearly stated
- High-throughput sequence data should be uploaded before submission, with a private link for reviewers provided (these are available from both GEO and ArrayExpress)

Please outline where this information can be found within the submission (e.g., sections or figure legends), or explain why this information doesn't apply to your submission:

Each experiment presented in the paper was repeated in multiple independent samples / animals. All attempts at replication were successful. We did not exclude any data from consideration.
The investigators were not blinded during data collection. Blinding during analysis was not necessary because the results are quantitative and did not require subjective judgment or interpretation. Whenever possible, quantifications were performed using automated pipeline(s) applied equally to all conditions and replicates for a given probe. For the described experimental designs, therefore, blinding is typically not used in the field.
Antibody validation: the antibody used – monoclonal anti-actin, α-smooth muscle-FITC (clone 1A4) – is commercially available from MilliporeSigma (catalog number F3777). This monoclonal α-SMA-FITC antibody has been validated by the manufacturer (ELISA and immunoblotting). In addition, this antibody was also validated in previously published work (Rønnov-Jessen and Petersen, The Journal of Cell Biology (1996) 134 (1); Grubb et al., Nature Communications (2020) 11:395; Weiss et al., Development (2014) 141(17); etc.).

Statistical reporting

- Statistical analysis methods should be described and justified
- Raw data should be presented in figures whenever informative to do so (typically when N per group is less than 10)
- For each experiment, you should identify the statistical tests used, exact values of N, definitions of center, methods of multiple test correction, and dispersion and precision measures (e.g., mean, median, SD, SEM, confidence intervals; and, for the major substantive results, a measure of effect size (e.g., Pearson's r, Cohen's d)
- Report exact p-values wherever possible alongside the summary statistics and 95% confidence intervals. These should be reported for all key questions and not only when the p-value is less than 0.05.

Please outline where this information can be found within the submission (e.g., sections or figure legends), or explain why this information doesn't apply to your submission:

Relevant information is found in the materials and methods section (subsection data analysis). Raw data is shown in figures whenever appropriate (n < 10) and possible as individual dots. Results are presented as means ± SEM or ± SD (as specified in both the main text and legends). Statistical analyses were performed using paired or unpaired *t*-tests, one-way ANOVA with Tukey's HSD *post hoc* test or the Fisher Exact test (as dictated by data distribution and experimental design). Tests and the exact corresponding *p*-values that report statistical significance (≤0.05) are individually specified in the legends.
Data were analyzed offline using FitMaster 2.9 (HEKA Elektronik), IGOR Pro 8 (WaveMetrics), Excel 2016 (Microsoft, Seattle, WA), and Leica LAS X (Leica Microsystems) software. Dose-response curves were fitted by the Hill-equation. Time-lapse live-cell imaging data displaying both Ca^{2+} signals and tubular contractions were analyzed using custom-written code in MATLAB (The MathWorks, Natick, MA). Rendering and three-dimensional reconstruction of fluorescence images was performed using Imaris 8 microscopy image analysis software (Bitplane, Zurich).

(For large datasets, or papers with a very large number of statistical tests, you may upload a single table file with tests, Ns, etc., with reference to sections in the manuscript.)

Group allocation

- Indicate how samples were allocated into experimental groups (in the case of clinical studies, please specify allocation to treatment method); if randomization was used, please also state if restricted randomization was applied
- Indicate if masking was used during group allocation, data collection and/or data analysis

Please outline where this information can be found within the submission (e.g., sections or figure legends), or explain why this information doesn't apply to your submission:

N/A. Samples were not randomized across experiments. All experiments involved inbred mice in which comparisons were between animals of the same strain, differing only by genotype.

Additional data files ("source data")

- We encourage you to upload relevant additional data files, such as numerical data that are represented as a graph in a figure, or as a summary table
- Where provided, these should be in the most useful format, and they can be uploaded as "Source data" files linked to a main figure or table
- Include model definition files including the full list of parameters used
- Include code used for data analysis (e.g., R, MatLab)
- Avoid stating that data files are "available upon request"

Please indicate the figures or tables for which source data files have been provided:

Previously unpublished source code for data analysis in MATLAB (The MathWorks, Natick, MA; quantification of tubular contractions, flow strength/change, calcium signals) is available at: https://github.com/rwthlfb/Fleck_Kenzler_et_al
If not available within the paper, all raw and processed data that support the findings of this study are available from the corresponding author upon reasonable request.
